# Insights on the Effects of Resveratrol and Some of Its Derivatives in Cancer and Autoimmunity: A Molecule with a Dual Activity

**DOI:** 10.3390/antiox9020091

**Published:** 2020-01-22

**Authors:** Elena Gianchecchi, Alessandra Fierabracci

**Affiliations:** 1 VisMederi srl, Strada del Petriccio e Belriguardo, 35, 53100 Siena, Italy; elegianche@yahoo.it; 2 Infectivology and Clinical Trials Research Department, Children’s Hospital Bambino Gesù, Viale San Paolo 15, 00146 Rome, Italy

**Keywords:** resveratrol, cancer, autoimmunity

## Abstract

In recent years, the interest in natural compounds exerting immunoregulatory effects has enormously increased. Among these, the polyphenol resveratrol, found in a variety of foods and beverages, including red grapes and red wine, has been demonstrated to exert both in vitro and in vivo biological activities. More specifically, it has antiaging, cardioprotective, antioxidant, immunomodulatory, anti-inflammatory and chemopreventive activities. Due to its anti-proliferative, pro-apoptotic and immunoregulatory effects, resveratrol has gained substantial attention for the treatment of cancer or autoimmunity, which represent frequently diagnosed diseases with important consequences for the health of the patients affected. The aim of the present review is to focus on the role of resveratrol in the modulation of cancer as well as of several organ-specific or systemic autoimmune diseases, including autoimmune hepatitis, type 1 diabetes mellitus, inflammatory bowel disease, rheumatoid arthritis, systemic lupus erythematosus and multiple sclerosis.

## 1. Introduction

Traditional medicine, often recognized as complementary, alternative, or nonconventional medicine [1,2], relies on the use of bioactive natural compounds [3]. Many of these compounds are well known in scientific literature since they are traditionally used by various cultures worldwide [3,4].

In this regard, several classes of agents exerting anti-mutagen and anti-carcinogen activities have been identified in foods and beverages of natural origin [5]. A wide variety of polyphenolic compounds, described especially in vegetables and fruits (in particular in grapes and their derivatives), have attracted attention for their beneficial health properties as promising antitumor agents [6].

Resveratrol (trans 3,5,4′-trihydroxystilbene) is a natural non-flavonoid polyphenol found in its trans isomer form produced by many different plant species, such as red grapes varieties [7], peanuts [8], blueberries [9], and rhubarb [10]. This compound plays several biological activities having anticancer [11,12], antimicrobial [13,14], antioxidative [15], anti-neurodegenerative [16], anti-inflammatory and immunomodulatory properties in vitro and in vivo [17,18] and estrogenic effects in vitro [19]. Indeed, this polyphenol exerts a role towards adverse conditions including environmental stress, injury, or pathogenic attack, i.e., UV irradiation and fungal infection [20]. 

Firstly identified by Takaoka from white hellebore (*Veratrum grandiflorum*) in 1939, resveratrol was afterwards considered as a phytoalexin [21]. Remarkably, in 1992, Siemann [22] discovered its presence in red wine. This result was later used to explain the so-called “French Paradox”, referring to the low rates of coronary heart disease mortality in some areas of France, despite the elevated intake of fat and cholesterol through the daily diet of residents [23]. In 1997, Jang and coworkers discovered that resveratrol could prevent cancer development [24]. Several benefits from resveratrol are attributed to its antioxidant properties although its cardioprotective, immunomodulatory, anti-inflammatory and chemopreventive activities rely on other mechanisms [24]. 

Regarding the antioxidant potential of resveratrol, this compound is both a free radical scavenger as well a strong antioxidant due to its ability to induce the activities of numerous antioxidant enzymes [25]. In particular, the antioxidant capacity of polyphenolics is attributed to the redox properties of their phenolic hydroxyl groups and the potential for electron delocalization across the chemical structure [26]. Over the last ten years, several studies have highlighted the critical role played by reactive oxygen species (ROS) in arbitrating the development of oxidative stress [27]. Whereas reduced levels of ROS synthesis allow the maintenance of physiological functions, such as proliferation, signal transduction, gene expression and host defense [27], an excess in ROS accumulation could be harmful due to the oxidative changes affecting cellular macromolecules (lipid, proteins and nucleic acids) characterized by detrimental potential. 

Indeed, deoxyribonucleic acid (DNA) injury by ROS, which can cause potential single- and double-stranded DNA breaks by reacting with the nitrogenous bases and the sugar phosphate backbone, has been correlated with mutagenesis, oncogenesis and aging [28]. Among the oxidative lesions in DNA there are not only strand breaks but also base modifications, sugar damage, and abasic sites [29]. Due to the ability of oxidants, antioxidants and other determinants of the intracellular redox state to modulate gene transcription, ROS changes within mammalian cells can also modify the expression of numerous mammalian genes, such as oncogenes and amyotrophic lateral sclerosis-linked genes during transcription, as recently demonstrated by Li [30].

In the last years, there has been quick progress in clarifying the molecular mechanisms responsible for the antitumor effects of resveratrol [31]. More specifically, the chemopreventive ability of resveratrol has been linked to its ability to halt the activation of different carcinogens and/or to promote their detoxification, to avoid oxidative damage of target cell DNA, to lower inflammation and to reduce the proliferation of tumor cells [31,32]. The chemotherapeutic potential of resveratrol both in vitro and in vivo is sustained by the inhibition of angiogenic and metastatic processes of cancer progression and attenuation of chemotherapy resistance [33]. Resveratrol promotes the apoptosis of various premalignant or cancerous cells by regulating or inhibiting multiple pathways including the PI3K/Akt/mTOR and the mitogen-activated protein kinase pathways (MAPK) [34]. These abilities can underlie its chemopreventive and chemotherapeutic potential.

Approximately 18 years ago, resveratrol was proposed as an immune modulator able to regulate both innate and adaptive immune responses through interaction with several molecular pathways [35]. Indeed, experimental evidence in cell lines and animal models has demonstrated the immunomodulatory activity of resveratrol with dose-dependent opposite effects. More specifically, the compound acts as an immunosuppressive when administered at high doses, whilst at low doses it stimulates the immune system [36].

The effect of the molecule has been observed on several immunotypes producing macrophage, T cell and natural killer (NK) cell activation as well as being involved in the suppressive function of CD4+CD25+ regulatory cell subsets [35,37]. The molecule has also effects on B cell proliferation and autoantibodies production [17,38].

As reported by several in vivo studies conducted in animals and humans, resveratrol is characterized by a low oral bioavailability [39] due to a very limited intestinal uptake of the molecule [40]. This leads to minimum amounts in the bloodstream because of the extensive metabolism occurring in the gut [41] and liver [42]. The short initial half-life of the primary molecule is essentially caused by its rapid metabolism. The bulk of an intravenous dose of resveratrol is processed in sulfate conjugates in just 30 minutes in humans [43]. Several other natural stilbenoids, as well as those derived by the synthetic modification of the stilbene scaffold [44,45,46], present a similar structure to resveratrol, linked to its potential metabolism. They could have some of the beneficial properties of the parent molecule and produce even larger benefits [47]. 

Studies of these derivatives have constituted the basis for the development of novel resveratrol analogues through specific changes of the stilbene scaffold-achieving molecules with a stronger antitumor effect or other properties. The substitution of the hydroxyl groups with methoxyl groups meaningfully enhances resveratrol bioavailability by boosting its intestinal absorption and increasing hepatic stability [47]. For this reason, different methoxylated analogues of resveratrol have been formulated with the purpose of obtaining novel agents for cancer chemoprevention.

Along with resveratrol, several other stilbenes, with a similar structure to resveratrol, are naturally present in food. For example, pterostilbene (trans-3,5-dimethoxy-4’-hydroxystilbene), which is a structural analog of resveratrol generated by the substitution of two hydroxyl groups with two methoxyl groups, has been found in blueberries and has been studied most widely [48]. A higher lipophilicity of pterostilbene over resveratrol has been obtained through the substitution of hydroxyl with methoxyl groups resulting in better bioavailability. These differences in pharmacokinetics might account for the higher biological activity of pterostilbene over the parental compound resveratrol [48]. Pterostilbene, as resveratrol, is also not toxic when used at high dosages in humans [49].

Pterostilbene has been reported to exert various pharmacologic effects, including anticancer, anti-proliferative, pro-apoptotic, antioxidant, anti-inflammatory, anti-invasive and antimetastatic activities [48]. Pterostilbene has also been demonstrated to act specifically against tumor cells without triggering any acute toxicity to normal cells (reviewed in [50]).

**Figure 1:**
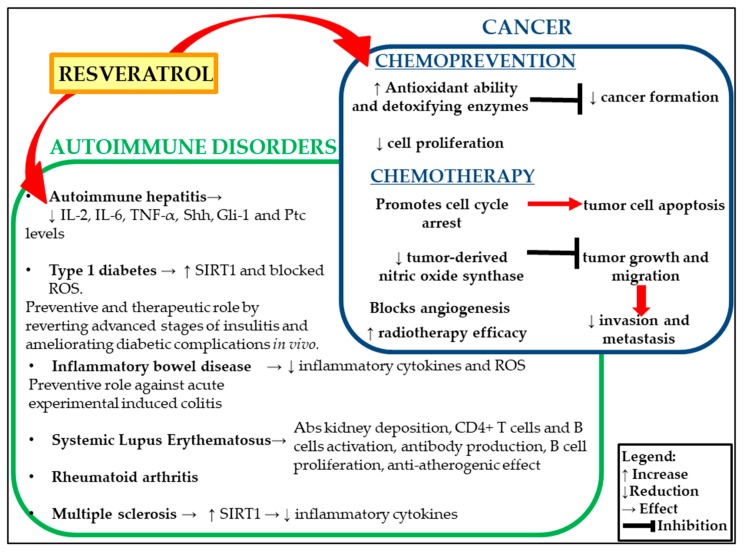
Dual role of resveratrol on autoimmune disorders and cancer.

In this review, we summarized the most recent updates on the effects of resveratrol in the management of cancer and chronic inflammatory conditions, such as autoimmune diseases (Figure 1).

## 2. Resveratrol and Cancer

During the development of cancer, there is a complex orchestrated progression wherein, due to genetic mutations, normal cells do not stop to grow, invade normal tissues and metastasize. Despite considerable efforts to find a treatment, this pathologic condition constitutes one of the most frequently diagnosed diseases whose morbidity and mortality represents a significant health issue globally. More specifically, it is the second cause of death in the world, having caused 9.6 million deaths in 2018 [51]. 

The ability of the compound to influence one step of this process attracts considerable attention since it could putatively represent an anticancer drug, which could be used in clinical settings [34]. 

For decades, the attention towards resveratrol has increased for its role in preventing and treating numerous pathologies, such as tumor, neurodegenerative, cardiovascular, inflammatory, and even autoimmune diseases. A growing amount of research has demonstrated that resveratrol induces cytotoxicity towards cancer both in vitro and in vivo and plays a chemopreventive role. 

The properties of resveratrol so far identified can be summarized as the following: resveratrol is able to induce the block of cell cycle causing the apoptosis of cancer cells, through the downregulation of tumor-derived nitric oxide synthase; furthermore, it halts the growth of cancer cells and their migration; its antioxidant ability avoids DNA damage responsible for tumor formation; and it regulates nuclear factor NF-κB activation [52]. 

Resveratrol has antitumor activity in different human cancers such as hepatocellular carcinoma [53] and ovarian carcinoma [54]. Recently, Zhong and colleagues [55] investigated the anti-tumoral effect of resveratrol in vivo in a rat orthotopic ovarian cancer (OC) model reporting that its intraperitoneal administration halted cancer cell proliferation without affecting normal tissues. In greater detail, the blockade of glycolysis and inhibition of AMPK/mTOR signaling induced by resveratrol were responsible for its antitumor activity in ovarian cancer cells [56]. Recently, Zhang and colleagues [57] reported for the first time that resveratrol promoted not only apoptosis but also immunogenic cell death of human and murine ovarian carcinoma cells.

In addition, resveratrol has been extensively investigated for its ability to enhance cell killing by radiation [58] and ionizing radiation (IR) mediated apoptosis in cancer cells [59].

On a general ground, the cellular effects of polyphenols, such as anti-proliferative and pro-apoptotic actions, have been recently correlated with their ability to modify the activity of topoisomerases [60]. These ubiquitous nuclear enzymes regulate the topological state of DNA by breaking and resealing one or both strands of a DNA duplex. Whereas lower eukaryotes, such as yeast and Drosophila, possess a single type II topoisomerase, eukaryotic cells have type II topoisomerase (Topo II) isoforms α and β. These two isoforms act in the principal cellular processes, engaging DNA by generating intermediate cleavable or covalent complexes with a short half-life [61]. Topo II plays a fundamental role in the survival of all eukaryotic cells constituting both an enzyme and a structural component of the nuclear matrix. It modulates the topological states of DNA by transient cleavage, strand passing and re-ligation of double-stranded DNA resulting in catenation, decatenation, knotting or unknotting of DNA molecules and relaxation of supercoiled DNA [62]. In addition, Topo II has a critical function in DNA replication and is necessary for condensation and segregation of chromosomes. Topo II expression depends on the cell cycle and its protein levels and catalytic activity reach a peak at G2/M. Topo II phosphorylation/dephosphorylation was supposed to constitute one of the regulatory checkpoints at the entry and progression of mitosis [61]. Topo II represents the molecular target of resveratrol, as supported by a growing number of studies [63,64] and elucidated recently by Lee and colleagues [60]. Recently, resveratrol was found to antagonize the expression of checkpoint genes and proliferative genes induced by thyroxine in human oral cancer cells [65].

Furthermore, resveratrol coadministration with other chemotherapeutic drugs was deeply helpful for cancer therapy in vitro and in vivo. Resveratrol and 5-fluorouracil coencapsulation in liposomal nanocarriers showed a higher cytotoxicity compared to the free drug combination when tested in vitro on head and neck cancer cell lines [66]. Doxorubicin (DOX) and resveratrol co-treatment overcame drug resistance by promoting the deregulation of the cell cycle and apoptosis in a B16/DOX murine melanoma model, lengthening mice survival compared to the untreated counterparts [67]. In a glioma nude mouse model, the combination of resveratrol with temozolomide (TMZ) potentiated the therapeutic efficacy of the latter both in vitro and in vivo [68]. More specifically, the combined treatment promoted apoptosis as well as cell differentiation and blocked the metastatic process by consistently inhibiting cell migration. Yuan [68] observed in fact that the therapy was able to induce glial fibrillary acidic protein (GFAP) expression, a marker characterizing differentiated astrocytes lost frequently with increasing grade of malignancy. Furthermore, the expression of matrix metalloproteinase-9 (MMP-9), which is an extracellular protease having a role in cell migration across basement membranes, was reduced. The recent study conducted by Zeng [69] demonstrated that resveratrol tumor necrosis factor-related apoptosis-inducing ligand (TRAIL) combination treatment was able to sensitize renal cell carcinoma (RCC) cells to TRAIL-induced death in vitro. When the co-treatment was administered in nude mice, the RCC xenograft growth was considerably reduced. In adriamycin-resistant leukemia K562/RA cells, when resveratrol was co-administered with arsenic trioxide (ATO), the apoptotic effect of the latter was potentiated. In addition, resveratrol diminished the toxicity of ATO alone.

In breast cancer cells, pterostilbene induces apoptosis in a caspase-dependent manner. More specifically, effector caspase-3 and -7 activation could be due to the loss of mitochondrial membrane potential and to the generation of superoxide anions. In the same way, caspase-dependent apoptosis upon treatment with pterostilbene is correlated with ROS generation, loss of mitochondrial membrane potential, cytochrome c release, a shift in the balance of pro- and anti-apoptotic B-cell lymphoma-2 (Bcl-2) proteins, and activation of caspases in a human gastric carcinoma cell line [70]. Furthermore, pterostilbene inhibits cell proliferation and cell cycle progression in a concentration- and time-dependent manner. For this purpose, cell cycle progression is blocked by pterostilbene at the G1 phase, associated by an increase in p53, p21, p27 and p16 proteins and a concomitant reduction in cyclin A, cyclin E and cyclin-dependent kinase (Cdk)2, Cdk4 and Cdk6 [70].

The coadministration of resveratrol and pterostilbene was able to synergistically inhibit the growth of triple-negative breast cancers (TNBC), representing about 10%–20% of total breast cancer. This was associated with a reduction in silent information regulator 1 (Sirtuin 1, SIRT) expression, a type III histone deacetylase (HDAC) involved in many molecular events, such as cancer and immune tolerance including the maintenance of peripheral T cell tolerance, and DNA methyltransferases (DNMTs) enzymes [71]. A subsequent study conducted by the same group [72] revealed that resveratrol/pterostilbene co-treatment was able to restore Estrogen Receptor-α (ERα) expression in ERα-negative breast cancer cells, characterized by a higher aggressivity and no response to conventional hormone-directed therapies. The study confirmed the role played by nutritional factors as regulators of gene expression. Furthermore, the use of these plant-based dietary compounds in combinatorial treatment allows one to overcome the side effects caused by conventional therapies used to reestablish ERα expression. The encapsulation of resveratrol into oxidized mesoporous carbon nanoparticles (oMCNs) could increase the solubility of resveratrol and improve the in vitro release property, leading to greater cytotoxic and pro-apoptotic effects [73]. The recent study conducted by Thipe [74] demonstrated that the use of resveratrol-conjugated gold nanoparticles improved resveratrol bioavailability and was effective against breast (MDAMB-231), pancreatic (PANC-1) and prostate (PC-3) tumor cell lines. 

In several studies, the 3,4,5,4’-trans-tetramethoxystilbene (TMS) was reported as the most potent pro-apoptotic analog of resveratrol (reviewed in [75]). 

TMS was also able to promote both apoptosis and autophagy in gefitinib-resistant (G-R) non-small-cell lung carcinoma (NSCLC) cells, whereas any inhibitory activity was exerted on other NSCLC cells and normal lung epithelial cells [76]. The mechanisms in resveratrol and its derivatives played their effects on NSCLC were elucidated by Lu and colleagues, who demonstrated that trans-3,5,4’-trimethoxystilbene diminished gefitinib resistance in NSCLCs [77], revealing that it occurred through the inhibition of the MAPK/Akt/Bcl-2 pathway by upregulating miR-345 and 498. A study conducted by Stivala et al. [78] described the stronger antioxidant effect of resveratrol compared to 3,5,4’-trimethoxystilbene. In addition, 3,5,4’-trimethoxystilbene was proven considerably more potent in inhibiting angiogenesis than resveratrol. When 3,5,4’-trimethoxystilbene was used, intersegmental vessel regression and downregulation of vascular endothelial growth factor receptor 2 (VEGFR2) mRNA expression were observed in zebrafish; in addition, 3,5,4’-trimethoxystilbene caused G2/M cell-cycle blockade in endothelial cells of zebrafish embryos. In the regulation of neovascularization in neoplasia two principal mechanisms were identified: first, anti-angiogenic compounds block neovascularization, and second, vascular-targeting agents disrupt immature vessels [79]. It has been hypothesized that 3,5,4’-trimethoxystilbene could act as an anti-angiogenic and anti-vascular compound by reducing VEGFR2 expression and inducing cell-cycle arrest at G2/M phase [79]. In regards to the antiangiogenic and vascular-targeting activity, Belleri et al. [80] reported that 3,5,4’-trimethoxystilbene has up to 100 times higher potency compared to the parent compound resveratrol; this was assessed through endothelial cell proliferation, sprouting, collagen gel invasion and morphogenesis. The vascular-targeting of 3,5,4’-trimethoxystilbene was due to the destabilization of microtubules, representing a target of numerous cancer chemotherapeutic agents, and depolymerization of tubulin. In addition, 3,5,4’-trimethoxystilbene was able to interfere with the microtubule organization center and with the migration of the endothelial cell [80]. Moreover, 3,5,4’-trimethoxystilbene inhibited human lung adenocarcinoma cell invasion by suppressing phosphorylation of stress-activated kinases (SAPK)/c-Jun N-terminal kinase (JNK) and p38 MAPK signaling pathways. This effect was associated with a reduction in nuclear factor-kappa B (NF-kB) and activator protein-1 (AP-1) proteins at nuclear level, representing two transcription factors implicated in invasion promotion. NF-kB and AP-1, in turn, induced the downregulation of matrix metalloproteinase (MMP)-2 expression [80]. Both 3,5,4’-trimethoxystilbene and resveratrol halted the migratory and invasive properties of HepG2 and Hep3B hepatocellular carcinoma cells following exposure to phorbol 12-myristate 13-acetate (PMA) and of PMA-untreated Hep3B cells. This anti-invasive effect was associated with a reduction in MMP-9 and MMP-2 activity, while tissue inhibitors of metalloproteinase (TIMP)-1 and TIMP-2 protein expressions increased [81].

Another limit of resveratrol is its bioavailability, characterized also by an elevated range of interindividual variation in a sex-independent manner [48]. The intake of low doses of resveratrol generates a maximum peak plasma concentration in the first 30 min, whereas at high doses the maximum peak plasma concentration is reached in 1.5–2h. Studies conducted in rodents demonstrated that resveratrol was eliminated rapidly at all tested doses of 5, 10, and 25mg/kg and the half-life time was only about 2h [82]. Such characteristics of resveratrol metabolism suggest that the proper way for obtaining optimal anti-tumoral effects could be a continuous drip or multiple oral administrations.

Compared to resveratrol, 3,4,5,4’-tetramethoxystilbene (DMU-212) exhibited increased pharmacological and pharmacokinetic properties with a higher metabolic stability and bioavailability [83]. In addition, DMU-212 presented a stronger inhibitory activity on the proliferation of melanoma cancer cells [84].

The putative anticancer effects of resveratrol have been also investigated in different leukemia cell lines. Indeed leukemia represents a heterogeneous group of diseases, encompassing acute myeloid leukemia, acute lymphoblastic leukemia, chronic myeloid leukemia and chronic lymphoblastic leukemia, which can be further classified into different subtypes. 

Bernhard et al. [85] demonstrated that resveratrol at 20 μM and higher doses arrests, in a concentration-dependent manner, the cell cycle in S-phase and apoptosis of T cell-derived T-ALL lymphocytic leukemia cell line CEM-C7H2. In addition, whereas the blocking of Fas or Fas Ligand (FasL) as well as the constitutive expression of cytokine response modifier A (CrmA) (an effective inhibitor of the Caspase family other than Caspase-6) did not impact resveratrol-induced apoptosis in CEM-C7H2, the treatment with z-IETD-fmk, an inhibitor of Caspase-6, almost totally halted the pro-apoptotic effect of resveratrol [85]. Additionally, resveratrol considerably and irreversibly blocked the growth of human chronic myeloid (K562) and acute lymphoblastic (HSB-2) leukemia cells; moreover, this effect correlated with a marked apoptosis. The sensitivity to resveratrol-induced apoptosis of the two cell lines investigated was associated with both Bax-increased expression and the release of cytochrome c [86]. Moreover, resveratrol inhibited growth and promoted both apoptosis and cell cycle arrest at G1 phase in a mouse lymphocytic leukemia L1210 cell line. A dose-related regulatory activity on both innate and specific immune functions to L1210 bearing mice, the increase of lymphocyte proliferation and NK activity, the normalization of CD4/CD8 ratios, a reduction in Interleukin-6 (IL-6) release and content have been observed. In particular, this last finding has been hypothesized to represent, at least in part, the mechanism of the antitumor immune activity of resveratrol [68]. In fact, this intracellular cytokine has been implicated in tumorigenesis constituting a paracrine growth factor in several cancers [87].

Similar effects have been observed in vitro also in human leukemia cell types, although the compound’s mechanism of action remains yet to be clarified. Resveratrol-induced cell death of human promyelocytic leukemia (HL-60) cells was due to the proteolytic cleavage of caspase substrate poly (ADP-ribose) polymerase (PARP) and depended on CD95-signaling [88]. HL60 leukemia cells were induced to apoptosis by resveratrol in a dose-dependent manner at concentrations ranging from 8 to 32 μM, via promoting CD95−CD95L interaction on the cell surface [88], creating a death-inducing signaling complex (DISC) at the cytoplasmic CD95 receptor, inducing the release of cytochrome C from mitochondria, and activating Caspase-9 and downstream Caspase-3 in a mitochondrial-independent manner [89]. In addition, resveratrol was demonstrated not only to diminish cell viability as well as DNA synthesis, but the analysis of the cell cycle revealed that resveratrol enhanced the proportion of the subdiploid cell population in HL60 cells. Furthermore, resveratrol decreased the expression of Bcl-2, representing an anti-apoptotic protein whose role is the maintenance of mitochondrial membrane integrity [90]. This effect correlated with increased expression of the pro-apoptotic Bax protein [91], annexin A1, growth arrest-induced and DNA damage-induced gene 45α (GADD45α), and cleaved Caspase-3 [92]. Resveratrol used at a 100 μM dose leads to growth inhibition and proliferation arrest of HL60 cells by downregulating anti-apoptotic protein Bcl-2 expression and lowering the viability of DNA synthesis [92]. Besides, resveratrol induced FasL-related apoptosis of HL-60 cells through Cdc42 activation of apoptosis signal-regulating kinase 1 (ASK1)/c-Jun N-terminal kinase (JNK) dependent signaling pathway [93]. Further investigations regarding the mechanisms underlying the pro-apoptotic effect of the compound on HL60 cells reported de novo production and accumulation of ceramide by raising the expression of ceramide-generating protein longevity assurance, and decreasing that of anti-apoptotic sphingosine kinase-1 (SK-1) and glucosylceramide synthase [94]. These results regarding the pro-apoptotic role played by resveratrol are in agreement with previous data obtained from acute myeloid leukemia (AML) cells reporting the subsequent induction of DNA repair enzyme PARP (poly adenosine diphosphate (ADP)-ribose polymerase) cleavage due to cysteine protease Caspase-3 activation occurring upon treatment with the compound [95]. 

In addition to proliferation blockage and cell apoptosis promotion, resveratrol was able to induce differentiation of the human erythro-megakaryoblastic leukemia cell line K562 as demonstrated by Yan et al. [96]. In more details, they reported that resveratrol induced cell differentiation by favoring glycophorin A, HBA1, HBB and γ-globin expression, in particular when used at the concentration of 50 μM. Conversely, the expression of these four genes was lessened when resveratrol was administered at 100 μM. Although in many types of cancer resveratrol supplementation has shown positive results, in others effects have been ambiguous, as reported on androgen-responsive LNCaP human prostate cancer cells in vitro and in vivo [97], and sometimes there are even detrimental effects [98,99]. This is due to resveratrol’s mechanism of action, which depends on the intrinsic molecular properties of the cancer model under investigation. 

Even though resveratrol has shown a promising antitumor effect in different types of cancer, reverting also the multidrug resistance in tumor cells, and increasing the therapeutic outcomes of the standard chemotherapeutic agents, additional clinical studies are necessary before its application in clinical treatment. So far the majority of the clinical trials have had the aim of investigating the safety, bioavailability, pharmacokinetics and tolerability of resveratrol, whereas its potential anticancer properties have been evaluated only in a limited number of studies. In addition, no clinical trial based on the co-administration of resveratrol with other antitumor drugs has been performed or is currently in progress.

## 3. Resveratrol and Autoimmune Diseases

A direct consequence of the lengthening of human life expectancy is the aging of most of the population worldwide. Aging represents a risk factor for the onset of chronic diseases, including autoimmune disorders [100]. An elevated amount of evidence has reported that age-associated disturbances involving innate immunity (immune response of neutrophils and macrophages) and adaptive immunity (B cell and T cell development) have a higher prevalence in respect to young subjects [100], even though the onset of the latter can occur at different ages depending on the autoimmune condition. Besides conventional treatments such as analgesics, non-steroidal anti-inflammatory drugs and glucocorticoids, innovative therapies based on therapeutic immunosuppression and biological agents, as well as molecules derived from natural products, have been widely studied for their pharmacological effect on both organ-specific and systemic autoimmune disorders [101]. 

### 3.1. Autoimmune Hepatitis

Autoimmune hepatitis (AIH) represents an idiopathic inflammatory pathology affecting the liver which presents a loss of self-tolerance to hepatocyte-specific autoantigen, leading to the synthesis of autoantibodies and the production of dense lymphoplasmacytic inflammatory infiltrates in the portal tracts [102]. Activation and clone expansion of T cells lead to B cell release of autoantibodies, pro-inflammatory cytokines and hepatocyte destruction finally responsible for liver failure [103]. Zhou et al. observed the protective role played by resveratrol against concanavalin-A- (ConA-) induced liver injury in mice by significantly inhibiting IL-2, IL-6, tumor necrosis factor α (TNF-α), Sonic hedgehog (Shh), Glioblastoma- (Gli-) 1, and Patched (Ptc) levels, probably through the modulation of the hedgehog signal pathway [104]. 

### 3.2. Type 1 Diabetes Mellitus

Bertelli and colleagues [105] suggested the putative therapeutic application of resveratrol in type 1 diabetes mellitus (T1D) induced cerebrovascular dysfunctions upon the observation that rats affected by T1D had their vascular functions restored following long-term resveratrol treatment [105]. Yun and colleagues [106] explored the resveratrol mechanism of action by using human monocytes obtained from T1D patients, revealing that it induced the overexpression of SIRT1 and blocked the cellular oxidative stress. Resveratrol was demonstrated to have both preventive and therapeutic effects by reverting the advanced stages of insulitis affecting the islets of Langerhans in a non-obese diabetic (NOD) mice model [107]. This was due to a reduced expression of chemokine receptor 6 (CCR6) in T helper (Th17) cells and pathogenic CD11b+F4/80hi macrophages responsible for halting cell migration from peripheral lymphoid organs to the pancreas. A following study by Kaur [108] reported the amelioration of diabetic complications, as well as the loss of β-cells, pancreatic and hepatic oxidative stress in streptozotocin-induced diabetic rats treated with 25 mg/kg of resveratrol. When co-administered with insulin, resveratrol was able to improve glycemic control in diabetic rats, lowering the blood glucose to the levels reported in non-diabetic rats and cutting down glycosuria [109].

### 3.3. Inflammatory Bowel Disease (IBD)

A multifactorial origin has been hypothesized for inflammatory bowel disease (IBD) onset, encompassing Crohn’s disease (CD) and ulcerative colitis (UC). Among the factors implicated in its pathogenesis, genetics, bacteria, and an altered immune system response have been identified to play a promoting role for this autoimmune disorder [110]. More specifically, the impairment in the intestinal mucosal barrier allows the translocation of commensal bacteria as well as bacterial products into the intestinal wall, activating neutrophils and macrophages located in the epithelium. As a consequence of such activation, inflammatory mediators, including ROS and TNF-α, are released. The latter is synthetized especially by T lymphocytes upon antigen recognition. 

Resveratrol could diminish inflammatory cytokines and ROS in IBD animal models [111,112,113,114,115]. Furthermore, resveratrol pro-drugs, which have been developed to overcome the reduced bioavailability of resveratrol due to its rapid metabolic modification, lowered colon inflammation in a murine model [111]. This allowed the preservation of mucosal structure, promoted the presence of bifidobacteria and lactobacilli implicated in the maintenance of intestinal homeostasis and was correlated with an improvement in intestinal health [116]. Resveratrol also played a preventive role against the development of acute experimental-induced colitis when this compound was administered 48, 24 and 1 h before trinitrobenzene sulfonic acid (TNBS) intracolonic instillation [117]. 

Regarding the use of resveratrol in human clinical trials, to our knowledge, only the study conducted by Samsamikor et al. [118] investigated its effect in UC patients observing a diminishment of NF-kB activity in peripheral blood mononuclear cells (PBMCs), and of TNF-α and high sensitivity C-reactive protein (hs-CRP) in plasma.

### 3.4. Rheumatoid Arthritis

In an adjuvant-induced arthritis rat model, when used in association with curcumin through lipid-core nanocapsules, resveratrol showed a stronger effect in reducing paw oedema compared to resveratrol alone [119]. This promising result was subsequently confirmed by Riveiro-Naveira et al. [120] in an acute antigen-induced arthritis model wherein resveratrol was administered through the diet [120]. Promising results were also observed when the molecule was tested in gouty arthritis animal models. More specifically, sodium alginate and resveratrol co-treatment lowered IL-1β, CC-chemokine receptor 5 (CCR5) and CXC-chemokine ligand 10 (CXCL10) levels in the synovial tissue of a monosodium urate-induced mouse model of acute gouty arthritis. Further, at 6 hours after treatment, a marked improvement was observed in mice co-treated with sodium alginate and resveratrol compared to those receiving resveratrol alone [121]. A following study conducted by Chen et al. [122] reported a reduction in serum uric acid levels after resveratrol consumption by hyperuricaemic mice, allowing the hypothesis for its preventive use against recurrent attacks of gout [122].

### 3.5. Systemic Lupus Erythematosus

Resveratrol showed protective activity in a pristane-induced lupus mouse model. More specifically, it reduced proteinuria, IgM and IgG kidney deposition, and kidney histological lesions. In addition, the activation of CD4+ T cells and B cells was inhibited in vitro, and antibody generation and B cell proliferation halted [123]. Feng et al. [124] reported the vascular protective effect of resveratrol in the ApoE−/− Fas−/−C57BL/6 mice resembling SLE. This compound had anti-atherogenic properties inducing the increase of the cholesterol efflux pathway [125]. These data were also confirmed by Voloshyna et al. [126] reporting that resveratrol countered SLE-associated atherogenicity through the normalization of cholesterol efflux.

### 3.6. Multiple Sclerosis

Miyazaki et al. [127] proved that the induction of SIRT1 with resveratrol was able to normalize the altered synthesis of pro-inflammatory cytokines by multiple sclerosis (MS) B cells through the regulation of micro RNA (miR)-132 expression. In more detail, resveratrol not only considerably halted the aberrant lymphotoxin (LT) production, but could also inhibit TNFα synthesis by MS B cells, even in subjects characterized by the highest levels of B cell TNFα production. Conversely, no effect on IL-10 levels was observed. Hence, resveratrol represents a promising treatment for the reduction of inflammation in the central nervous system (CNS) as well as in the periphery and in the target organ of patients presenting relapsing and progressive forms of the illness. These data are in accordance with previous observations reporting the protective effect of resveratrol against experimental autoimmune encephalomyelitis (EAE) by activating multiple SIRT1 targets [128,129]. Contrasting results regarding the neuroprotective effect of resveratrol on EAE models were reported by Sato et al. [130]. In more detail, significant exacerbation of autoimmune and viral models of MS, without exerting any neuroprotective activity in the CNS in both models following resveratrol treatment, was observed [130]. It is also plausible that the contrasting effects reported for resveratrol on cytokine production in EAE may depend on the stage and course of disease, dose administered or the use of different antigens used to sensitize the animals in different mouse strains [130].

## 4. Conclusions

Due to its multiple beneficial properties, including not only cardio and neuroprotective properties but also antioxidant, anti-inflammatory and anti-tumoral effects, the attention on the use of this compound in different pathological conditions is increased. Efforts should be made to evaluate further the compound during clinical trials and to translate the positive results obtained from in vitro and in vivo experiments into the development of a therapeutic agent to be used also for prevention of the onset of different diseases.
